# When Psychosis Masks Neurodegeneration: A Case of Sporadic Creutzfeldt-Jakob Disease

**DOI:** 10.7759/cureus.116539

**Published:** 2026-09-19

**Authors:** Gayathri Jayakumar, Eraj Rahman, Radhiya Ms Bakth, Abdurahman Moalin, Mosab Yousif

**Affiliations:** 1 Gastroenterology and Hepatology, Newham University Hospital, Barts Health NHS Trust, London, GBR; 2 Acute Medicine, Newham University Hospital, Barts Health NHS Trust, London, GBR; 3 Geriatrics, Newham University Hospital, Barts Health NHS Trust, London, GBR

**Keywords:** cognitive decline, creutzfeldt-jakob disease, neurodegenerative disorders, prion disease, psychiatric symptoms

## Abstract

We present a diagnostically challenging case of a 57-year-old male with new-onset persecutory delusions, hallucinations, functional decline, and gait instability following recent travel. While psychiatric features are recognised in Creutzfeldt-Jakob disease (CJD), they are often underappreciated as initial manifestations, leading to misdiagnosis and delayed recognition. Initially suspected to have a primary psychotic disorder, the patient's rapidly progressive course, neurological signs, characteristic MRI findings, and positive cerebrospinal fluid (CSF) real-time quaking-induced conversion (RT-QuIC) confirmed a diagnosis of sporadic Creutzfeldt-Jakob disease (sCJD). This case underscores the need to consider organic and neurodegenerative etiologies in patients with acute psychiatric symptoms and gait disturbance.

## Introduction

Creutzfeldt-Jakob disease (CJD) belongs to a family of spongiform encephalopathies, which are rare fatal disorders that affect humans and several animal species alike [1]. What makes CJD particularly difficult to diagnose early is its resemblance to those of more familiar conditions such as psychiatric illness, autoimmune encephalitis, or the common dementias [2]. Globally, the disease affects roughly one to two people per million each year, and once symptoms begin, mean survival drops to less than a year [3].

Creutzfeldt-Jakob disease (CJD) is categorised etiologically into four main subtypes: sporadic, familial (genetic), iatrogenic, and variant forms [3,4]. Sporadic CJD (sCJD) arises spontaneously when the normal cellular prion protein (PrPc) undergoes conformational conversion into its abnormal isoform (PrPSc) through an unidentified mechanism [4,5]. It accounts for the majority of all CJD cases, with a mean age of onset around 65 years, and affects males and females equally [5]. The disease usually follows a rapidly progressive course, and most patients succumb to infection or cardiorespiratory failure within 12 months of symptom onset [3,5].

The neuropathological hallmarks of the disease are prion protein deposition, spongiform change in neural tissue, neuronal loss, and gliosis [6]. The prions carry no nucleic acid, which makes them a unique infectious agent. Instead, they consist of a misfolded version of an endogenous brain protein, the cellular prion protein (PrPc), which converts to a pathological isoform (PrPSc) rich in beta-sheet structure [5,6]. This abnormal form accumulates as amyloid within the central nervous system (CNS), causing the characteristic spongiform encephalopathy [5]. Human prion diseases are characterised by the histopathological triad of cortical spongiform change, neuronal cell loss, and gliosis [6].

CJD can occur through transmission via consumption of infected neural tissue or via inoculation [5]. This can be understood from the historical documentation of the disease associated with depth EEG electrodes, corneal grafts, dura mater transplants, and cadaveric growth hormone preparations. To make matters worse, the prion proteins are notably resistant to standard cooking and sterilisation methods [5]. A heritable form also exists, caused by mutations in the PRNP gene [5].

When psychiatric symptoms open the clinical picture, as they did in this patient, the underlying neurodegenerative process is easily overlooked. Mood changes, paranoia, and hallucinations are uncommon early features of CJD; hence, they tend to be attributed to primary psychiatric illness, especially when neurological signs have not yet surfaced [1,2]. One useful pointer in this setting is age: new-onset psychosis in a person in their mid- to late fifties, with no previous psychiatric history, is rarely schizophrenia and should prompt a thorough search for an organic explanation, especially when neurological signs coexist. This study aimed to present a middle-aged man with psychiatric symptoms and gait disturbance who was ultimately diagnosed as a CJD case.

## Case presentation

A middle-aged man with no prior medical or psychiatric history, and no history of organ transplantation, dura mater grafting, or exposure to cadaveric-derived materials, presented with a two-week history of behavioural change characterised by paranoia and auditory hallucinations following travel to Sri Lanka. He had departed the United Kingdom for Sri Lanka on Day 0 and returned on Day 17, and presented to the ED 3 days after return to the UK on Day 20.

Initial assessment, including routine blood investigations and CT brain imaging, was unremarkable; therefore, a psychiatric evaluation was conducted, which demonstrated no immediate risk to self or others; hence, he was discharged with an outpatient MRI request and follow-up under the Home Treatment Team (HTT). The outpatient MRI was requested because, despite the unremarkable initial investigations, the new rapid-onset psychiatric presentation without any prior history/family history raised concern for an underlying neurological or organic disorder. Whilst awaiting the MRI scan, he remained under HTT review and quetiapine 50 mg once daily was commenced for behavioural disturbance, and physiotherapy input was arranged in view of worsening mobility. He subsequently failed to attend his scheduled MRI on Day 57 due to progressive gait disturbance and ataxia. A repeat MRI appointment was arranged for Day 60; however, by this time his neurological deterioration had progressed, and he required hospital admission.

The patient reported no preceding psychiatric or neurological symptoms before travel, apart from mild low mood. Shortly after arrival in Sri Lanka, he developed gait unsteadiness accompanied by persecutory delusions and auditory hallucinations involving multiple threatening voices. These symptoms progressed rapidly, resulting in severe anxiety, fear for personal safety, and a fixed belief that others intended to cause him harm. Collateral history suggested that mild gait unsteadiness may have been present before travel; however, no psychotic symptoms were evident at that time. Following his return to the United Kingdom, there was progressive deterioration characterised by worsening confusion, agitation, and functional decline. He became increasingly housebound, with reduced oral intake and self-care, and developed a fixed delusional belief that relatives in Sri Lanka had subjected him to harmful ritual practices. A progressive and parallel evolution of neuropsychiatric symptoms was noted.

His speech was notably variable, ranging from marked paucity to intermittent spontaneous output. Cognitive assessment demonstrated profound impairment, with an Abbreviated Mental Test Score (AMTS) of 4/10 [7]. Formal Mini-Mental State Examination (MMSE) or Montreal Cognitive Assessment (MoCA) was not performed, as the patient's rapidly progressive cognitive and psychiatric deterioration made formal extended cognitive testing impractical. He was disoriented to person, time, and place, and attention, and exhibited deficits in short-term recall, while some autobiographical information and simple attention tasks remained intact. His neurological examination revealed a mask-like facies and polyminimyoclonus, and generalised hypertonia with paratonia was present. A vertical supranuclear gaze palsy was evident, while other cranial nerve functions remained intact. Cerebellar testing demonstrated dysmetria on finger-nose assessment, impaired heel-shin coordination, and dysdiadochokinesia. Ophthalmological examination showed no Kayser-Fleischer rings. He exhibited a broad-based gait and shuffling, and he required assistance for safe mobilisation.

The differential diagnosis included sporadic Creutzfeldt-Jakob disease (sCJD), progressive supranuclear palsy (PSP), autoimmune encephalitis, Wilson’s disease, and a primary psychotic disorder [2,4]. sCJD was considered due to the rapidly progressive cognitive decline, myoclonus, paratonia, gait disturbance, and prominent neuropsychiatric features. PSP was considered due to vertical gaze palsy, axial rigidity, and postural instability. Autoimmune encephalitis remained a consideration given the subacute onset of psychiatric and neurological features, and Wilson’s disease was considered in view of neuropsychiatric symptoms in a younger patient; however, normal ceruloplasmin, absence of hepatic dysfunction, and lack of Kayser-Fleischer rings made this diagnosis unlikely. A primary psychotic disorder was deemed less likely given the emergence of objective neurological signs and the rapidly progressive course.

Investigations

Initial laboratory investigations demonstrated mild lymphopenia, with otherwise normal inflammatory markers, including C-reactive protein. Vitamin B12, folate, thyroid function tests, renal profile, electrolytes, and calcium and magnesium levels were within normal limits. HIV testing was negative, and serum ceruloplasmin was normal. Contrast-enhanced brain CT showed no acute intracranial abnormality. MRI brain demonstrated diffusion-weighted and T2 hyperintensities involving the caudate nuclei, putamen, pulvinar, and dorsomedial thalami and characteristic hockey-stick sign, findings highly suggestive of prion disease (Fig. 1, 2).

**Figure 1:**
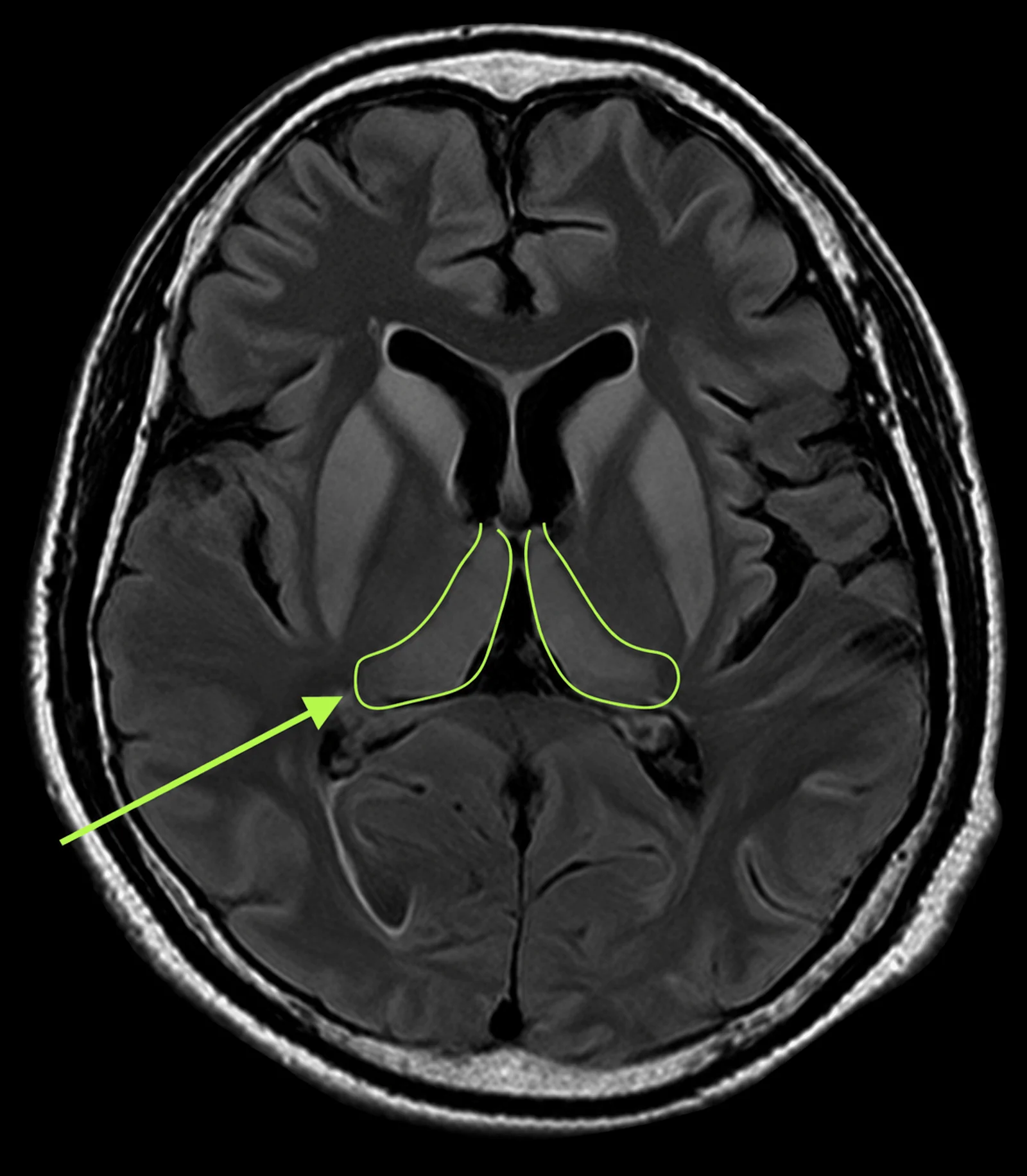
MRI brain T2 FLAIR MRI of the brain demonstrating characteristic features of sCJD. The green arrow indicates bilateral dorsomedial thalamic hyperintensity, producing the characteristic “hockey-stick” sign. sCJD: sporadic Creutzfeldt-Jakob disease; FLAIR: fluid-attenuated inversion recovery

**Figure 2:**
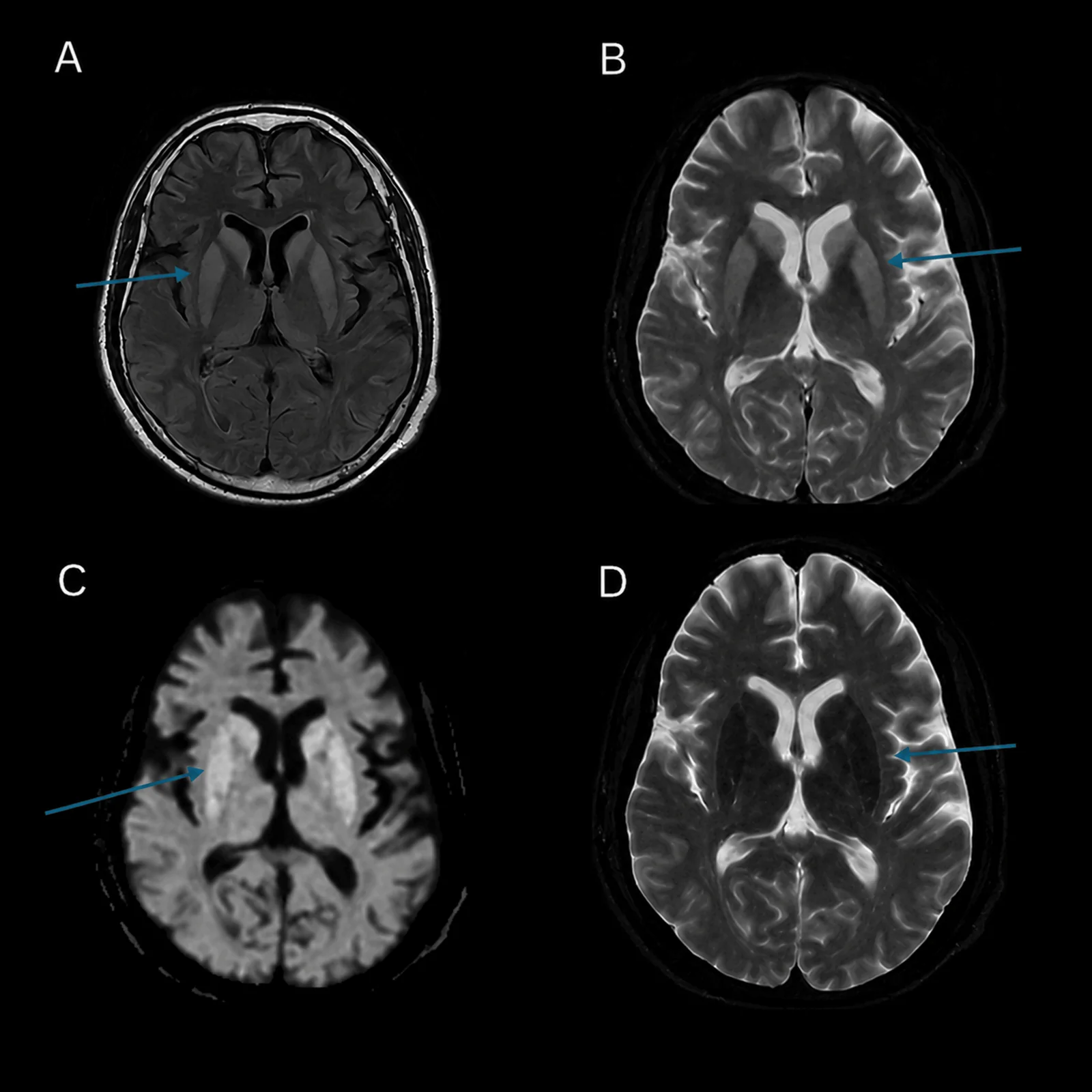
MRI Brain Hyperintense signal in the right and left heads of the caudate and putamen (blue arrows). A: T2 FLAIR; B: T2-weighted; C: diffusion-weighted imaging (DWI); D: apparent diffusion coefficient (ADC) map. The DWI sequence (C) demonstrates areas of high signal intensity, with corresponding low signal on the ADC map (D), confirming true diffusion restriction. FLAIR: fluid-attenuated inversion recovery

Cerebrospinal fluid (CSF) analysis was acellular, with normal protein and glucose concentrations. RT-QuIC assay was positive, suggesting the diagnosis of prion disease via the National CJD Surveillance Unit. CSF autoimmune and paraneoplastic panels, including NMDA receptor, LGI1, and CASPR2 antibodies, were negative. Additional investigations for HIV, hepatitis, vasculitis, and Wilson’s disease were unremarkable. EEG was not performed during the diagnostic work-up.

A multidisciplinary approach was adopted involving neurology, psychiatry, occupational therapy, speech and language therapy, and palliative care. Supportive management included venous thromboembolism (VTE) prophylaxis, vitamin supplementation with Pabrinex® (Haupt Pharma Wülfing GmbH, Gronau/Leine, Germany), physiotherapy, and nursing care. Despite comprehensive supportive measures, there was continued clinical deterioration. The patient was subsequently discharged to a nursing facility, where he died approximately one month later. The clinical timeline of the case is presented in Fig. 3.

**Figure 3:**
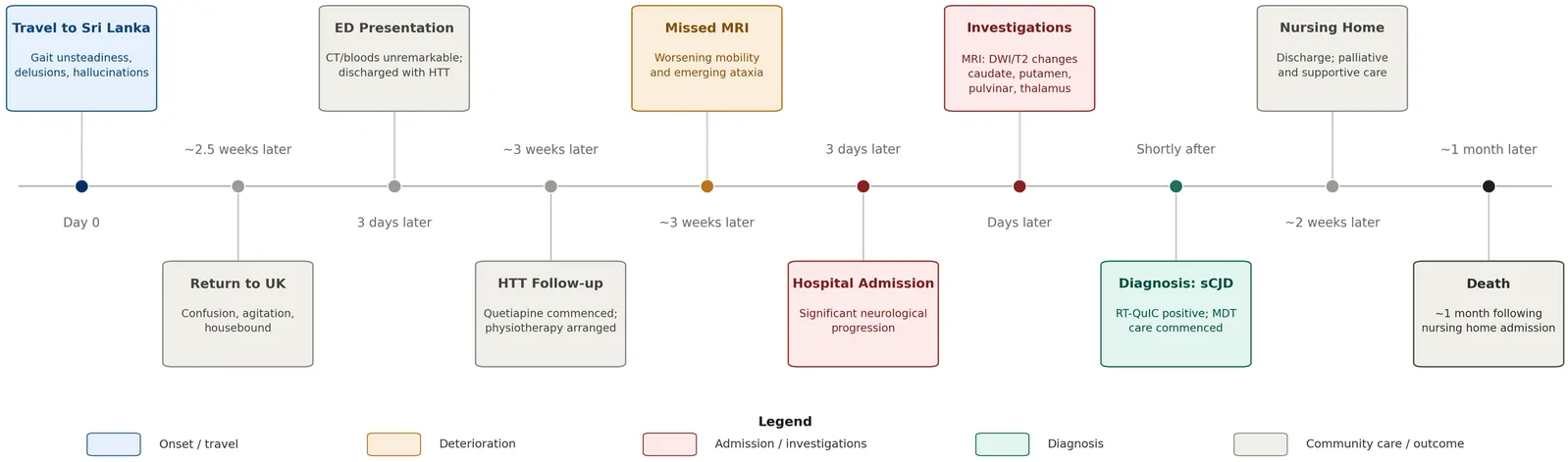
Clinical timeline of a middle-aged male presenting with rapidly progressive neuropsychiatric and neurological decline culminating in a diagnosis of sporadic Creutzfeldt-Jakob disease (sCJD). The timeline illustrates the clinical course from initial travel to Sri Lanka, during which psychiatric symptoms and gait unsteadiness first emerged, through to death approximately one month after nursing home discharge. Colour coding denotes clinical phase: blue (onset/travel), amber (clinical deterioration), red (inpatient admission and investigations), teal (confirmed diagnosis), and grey (community care and outcome). DWI: diffusion-weighted imaging; HTT: Home Treatment Team; MDT: multidisciplinary team; RT-QuIC: real-time quaking-induced conversion; SALT: speech and language therapy; sCJD: sporadic Creutzfeldt-Jakob disease.

## Discussion

This case highlights that psychiatric presentations of CJD are rare but do occur and should be considered in the differential diagnosis of new-onset psychosis, particularly when accompanied by neurological signs and rapid clinical progression. Although neuropsychiatric symptoms can occur during the course of sCJD, a predominantly psychiatric presentation is uncommon and may initially suggest a primary psychiatric disorder [8,9]. Although familial CJD can present with clinical features similar to sporadic CJD, the absence of a relevant family history in this patient made a familial form less likely, and genetic testing was therefore not pursued.

In this patient, the absence of a previous psychiatric history, middle age at presentation, and subsequent rapid deterioration were important diagnostic features. A first presentation of schizophrenia at this age would be highly unusual and should prompt consideration of an organic cause, particularly when neurological abnormalities are present. The subsequent development of gait disturbance, cognitive decline, and motor features further increased suspicion of an underlying neurological disorder.

Psychiatric manifestations in CJD can include delusions, hallucinations, mood disturbance, anxiety, and behavioural changes. Neuropsychiatric symptoms are common at some point during the course of sCJD and may occur early in the illness [8]. However, prominent psychiatric symptoms as the initial or predominant presentation remain uncommon. Unusual clinical presentations, including prominent psychiatric manifestations, can make the early diagnosis of CJD challenging [9]. This distinction is important, as neuropsychiatric symptoms occurring during the disease course are different from psychosis being the main presenting complaint.

The revised International CJD Surveillance Network diagnostic criteria focus on rapidly progressive neurological disease supported by characteristic clinical, MRI, EEG, or CSF findings, and psychiatric symptoms are not included as a core clinical feature [10]. This may contribute to diagnostic difficulty in patients whose initial presentation is predominantly psychiatric. In such cases, the emergence of neurological signs or rapid progression should prompt reconsideration of the initial psychiatric diagnosis and further neurological investigation.

Brain MRI is an important diagnostic investigation in suspected CJD, with reported sensitivity of approximately 90% [11]. Typical findings include diffusion restriction and T2/fluid-attenuated inversion recovery (FLAIR) hyperintensity involving the cortex and basal ganglia, particularly the caudate and putamen [2,11]. In this case, MRI demonstrated diffusion-weighted hyperintensity involving the basal ganglia and cortical regions, consistent with previously described findings in sCJD. CSF RT-QuIC subsequently provided diagnostic confirmation. Although CSF biomarkers such as 14-3-3 and tau may support the diagnosis, they lack specificity, whereas RT-QuIC has substantially improved the accuracy of antemortem diagnosis [12,10].

Electroencephalogram was not performed in this patient. Periodic sharp wave complexes are a characteristic finding that may develop as sCJD progresses, although their sensitivity is limited [13]. Therefore, EEG findings are supportive rather than essential for diagnosis, particularly when characteristic MRI and CSF findings are present.

The differential diagnosis of rapidly progressive psychiatric and neurological symptoms remains broad and includes autoimmune encephalitis, infectious and metabolic disorders, and other rapidly progressive neurodegenerative diseases. However, new-onset psychosis accompanied by neurological signs or rapid progression should prompt early neurological assessment and investigation for an organic cause, including CJD.

Molecular subtyping of sCJD is based on PRNP codon 129 polymorphism and the biochemical characteristics of disease-associated prion protein. Six major molecular subtypes are recognised: MM1, MM2, MV1, MV2, VV1, and VV2 [5]. Certain subtypes are associated with characteristic clinical and imaging patterns. In this case, the basal ganglia and thalamic involvement could suggest a VV2 phenotype; however, molecular characterisation was not performed, and therefore a specific subtype could not be assigned.

There is currently no effective disease-modifying treatment for CJD, and management remains supportive. Early recognition is nevertheless important to allow appropriate communication with the patient and family, multidisciplinary care, and timely involvement of palliative care.

Although psychiatric presentations of CJD are uncommon, this case demonstrates that they can be the initial clinical focus. New-onset psychosis in a middle-aged patient, particularly when accompanied by neurological signs, cognitive decline, or rapid progression, should therefore prompt consideration of CJD and early neurological investigation.

## Conclusions

Sporadic Creutzfeldt-Jakob disease should be considered in patients with acute psychiatric symptoms when accompanied by gait disturbance, myoclonus, or cognitive decline. Early neurological involvement, MRI, and CSF RT-QuIC testing are vital for timely diagnosis. Management remains supportive, highlighting the need for early palliative care engagement due to the rapid progression and fatal nature of the disease.

Although psychiatric presentations of sCJD are uncommon and are not currently incorporated into the international consensus diagnostic criteria for sporadic CJD, clinicians should consider CJD in the differential diagnosis of new-onset, rapidly progressive psychiatric symptoms, particularly when accompanied by evolving neurological signs and cognitive decline. Neuropsychiatric symptoms are common during the course of sCJD; however, they are less frequently the predominant presenting feature at disease onset, as occurred in our case, where psychiatric symptoms preceded the development of more characteristic neurological manifestations. MRI and CSF RT-QuIC are key diagnostic tools, and multidisciplinary care is essential in the management of sCJD.
